# A review of HIV-specific patient-reported measures of perceived barriers to antiretroviral therapy adherence: what themes are they covering?

**DOI:** 10.1186/s41687-019-0124-3

**Published:** 2019-06-27

**Authors:** Kim Engler, Isabelle Toupin, Serge Vicente, Sara Ahmed, Bertrand Lebouché

**Affiliations:** 1 Centre for Outcomes Research & Evaluation, Research Institute of the McGill University Health Centre, 5252 de Maisonneuve Blvd, Montreal, QC H4A 3S5 Canada; 2 School of Physical & Occupational Therapy, Faculty of Medicine, McGill University, 3654 prom Sir-William-Osler, Montreal, QC H3G 1Y5 Canada; 3 Department of Mathematics and Statistics, University of Montreal, 2920 chemin de la Tour, Montreal, QC H3T 1J4 Canada

## Background

In 2017, there were 2.2 million people living with human immunodeficiency virus (PLHIV) in western and central Europe and North America, with approximately 77% accessing antiretroviral therapy (ART) [1]. However, only 63% of PLHIV on ART are estimated to attain the ideal of at least 95% adherence [2]. Indefinitely maintaining ART adherence may be extremely difficult, given the numerous factors that can impede it [3]. While newer, more potent ART regimens may make perfect adherence less necessary [4], adherence difficulties are tied to a range of medically relevant psychosocial and structural issues. These include depression, alcohol/substance misuse, and health service-related barriers [5]. Indeed, regularly identifying a patient’s potential barriers to ART adherence is explicitly recommended in some HIV treatment guidelines [6]. Doing so could help address previously undetected problems and prevent virologic failures. Nevertheless, how best to do this remains less clear. Given the many recognized barriers to ART adherence, such an assessment could prove time-consuming [7].

Patient-reported outcome measures (PROM) could offer a solution and their use is growing in healthcare [8]. While published initiatives of their implementation in HIV care are few (e.g., [9, 10]), using them to screen for barriers prior to the clinic visit could offer a quick and affordable solution and lead to more patient-centered counseling and intervention [7]. Yet there may be few comprehensive HIV-specific self-report measures for capturing and succinctly scoring patient perceived barriers to properly taking ART in developed countries [11]. It is also unclear to what extent PLHIV participated in their creation, considering that patient involvement is deemed essential to a PROM’s content validity [12]. In a previous research phase, our team generated a conceptual framework of ART adherence barriers based on a synthesis of qualitative studies with PLHIV in developed countries, to design a new PROM for use in routine HIV care in Canada and France [13]. With this review, we seek to: 1) identify existing patient-reported measures of barriers to ART adherence used in developed countries, and 2) examine their coverage of this patient-informed conceptual framework.

## Methods

### Conceptual framework

Forty-one qualitative studies with adult PLHIV on barriers to ART adherence in developed countries were synthesized with thematic analysis to create our framework. It defines 6 broad interrelated themes under which are grouped 20 subthemes of barriers. Details on this framework are published elsewhere [13].

### Search strategy and inclusion criteria

On July 4, 2018, four databases were searched for patient-reported measures of barriers to ART adherence: EMBASE, MEDLINE, PsychINFO, and Health and Psychological Instruments. Searches were adapted to each database and targeted words in the abstract referring to: 1) HIV; 2) adherence; 3) barriers; and 4) antiretroviral therapy. The searches were limited to English-language publications from 1996 and human adults (18 or 19 years and older). The precise search strings used are available upon request. Duplicates of all identified records were eliminated. Then, the title and abstract of each record were screened and the full-texts of all potentially relevant records were examined. Records of conference abstracts and opinion articles were excluded. A tenth of deduplicated records and 15% of full-texts were reviewed by IT to calculate interrater reliability with Cohen’s kappa [14] and percent agreement. The references of retained full-texts were also searched.

Instruments (e.g., questionnaires, checklists, subscales) were included if they served to quantify perceived barriers to ART adherence. Specifically, eligible instruments allowed respondents to indicate factors that prevented them from taking the medication, as prescribed. Instruments also needed to be HIV-specific (i.e. designed or adapted for PLHIV), used in developed countries [15], based on patient report, and published in English no earlier than 1996, when combination ART became the new standard of care. If several versions of an instrument were found, only the most complete version was retained, unless item content differed meaningfully between them, in which case all were retained. Instruments with fewer than 3 items were excluded. They were also excluded if all relevant instrument items were not obtained, after contacting the author(s).

### Data extraction

We extracted the following information for each retained measure: instrument and/or study name, if appropriate; instrument items; publication or version year of the document from which the instrument items were extracted; number of items; author description of what the instrument measures; mention and form of patient involvement in its development; and first author and year of the research article publication affiliated with the measure. Based on Weiring et al. [16], patient involvement was defined as explicit mention of patient participation in either determining the outcome measured (e.g., in developing its framework or domains); generating items; and/or verifying content validity, including comprehensibility (e.g., through interviews).

### Analysis of thematic coverage

Our methods draw on the approach taken by O’Brien et al. [17]. To compare instrument items against our conceptual framework, we used content analysis [18], allowing for the creation of new themes to accommodate the items. We sought to map each item to the framework, using the qualitative analysis software, Atlas.ti (v8). Items could be coded for several subthemes. KE mapped all instrument items. IT mapped 10% of the items (*n* = 43) to calculate percent agreement on each item’s main subtheme. To assess coverage of the concept of barriers to ART adherence, instrument breadth (representation of all original framework themes) and depth (representation of all original subthemes) were evaluated. Coverage was expressed with means (i.e. average instrument breadth and depth) and proportions (e.g., percentage of (sub)themes represented). We did not consider the number of items representing each (sub)theme.

## Results

### Search results

**Fig. 1:**
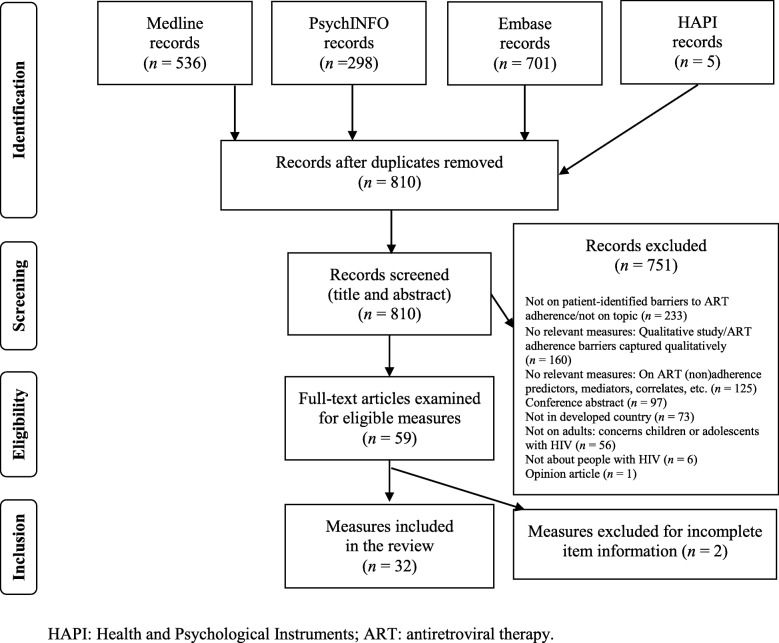
Search flow diagram

We reviewed a total of 1540 records, removing 730 duplicates (see Fig. 1). Following deduplication and exclusion of irrelevant records, based on title/abstract screening, the full-texts of 59 records were examined. Percent agreement was 90.1% for the deduplicated records and Cohen’s Kappa was 0.62, indicating substantial agreement [14]. Percent agreement for the full text articles was 88.9% and Cohen’s kappa was also 0.62. Relevant records and their references yielded 31 instruments for inclusion in the review. Two instruments were excluded [19, 20], given incomplete access to their items.

### Instrument description

Table 1 provides details on the instruments. Descriptions of an instrument could vary. All but one were described as measures of “reasons” (for “missing a dose”, “taking treatment breaks”, “nonadherence”, etc.) (*n* = 21) or “barriers” (to “adherence”, “taking antiretrovirals”, etc.) (*n* = 4) or both (*n* = 5). They originated from the Unites States (*n* = 20); Western Europe: Denmark, Germany, United Kingdom, and Sweden (*n* = 4); Australia (*n* = 3); Canada (*n* = 3); and Romania (*n* = 1). On average, they contained 13.5 items (*SD* = 5.8), with a range of 3 to 23. For 9 measures, patient involvement was reported. Its specified forms included interviews (*n* = 5), consultation (*n* = 3), and piloting/pretesting/pre-experimentation (*n* = 3). The version or publication year of the included instruments ranged from 1999 to 2017. An indication of their influence, authors reported adapting the Adult AIDS Clinical Trials Group (AACTG) adherence instruments [21] for 8 measures. Two original AACTG instruments were also included.

**Table 1:** Instrument characteristics (*n* = 31)

#	Instrument/study name (if appropriate)	First author of related publication	Version year	Country^b^	Description	Patient involvement		No. items^a^
						Yes	No	
1	Adult AIDS Clinical Trials Group (AACTG), Adherence Baseline Questionnaire II, 2001, Section C	Chesney et al. 2000 [21]	2001	USA	“Reasons why people may miss taking their medications”^c^		●	14
2	AACTG, Adherence Barriers Questionnaire, 2008, Question 2	Chesney et al. 2000 [21]	2008	USA	“Reasons why people may miss taking their medications”^c^		●	22
3	–	Amico et al. 2007 [22]	2007	USA	“Reasons for last having missed a dose of ART medication”		●	14
4	AACTG adherence instrument -Modified	Barfod et al. 2006 [23]	2006	Denmark	“Reasons for missing a dose”^c^	●		22
5	–	Boretzki et al. 2017 [24]	2017	Germany	“Reasons for nonadherence to antiretroviral therapy”		●	9
6	CEAT-VIH (Cuestionario para la Evaluacio’n de la Adhesio’n al Tratamiento Antirretroviral en Personas con Infeccio’n por VIH y Sida) -Romanian adaptation	Dima et al. 2013 [25] (based on Remor 2002)	2013	Romania	“Barriers to adherence” -“Antecedents of non-adherence behaviours”	●		3
7	–	Durante et al. 2003 [26]	2003	USA	“Reasons for missing doses of medication”^c^	●		14
8	–	Gifford et al. 2000 [27]	2000	USA	“Reasons for missing antiretroviral doses”		●	16
9	Questionnaire on Taking Antiretroviral Medication, Questions 3 and 4	Godin et al. 2003 [28]	2003	Canada	“Situations that might have hampered […] regular adherence to medication”	●		8
10	HIV Futures 3 Survey	Grierson et al. 2004 [29]	2004	Australia	“Reasons for taking treatment breaks”- Lifestyle and clinical reasons	●		16
11	HIV Futures 7 Survey	Grierson et al. 2013 [30]	2013	Australia	“Reasons for stopping ARV”	●		7
12	HIV Futures 7 Survey	Grierson et al. 2013 [30]	2013	Australia	“Reasons for taking breaks” -Lifestyle and clinical reasons	●		14
13	–	Harzke et al. 2004 [31]	2004	USA	“Perceived barriers to taking antiretrovirals” –“Forgetting to take medications” scale		●	3
14	HCSUS 2nd Follow-up, Section 4.5 Antiretroviral and Opportunistic Infection Medication	n.a.	1997	USA	“Reasons […] why you stopped taking this antiretroviral medication(s)”		●	11
15	–	Kalichman et al. 1999 [32]	1999	USA	“Perceived barriers to treatment and reasons for non-adherence”		●	9
16	–	Kalichman et al. 2017 [33]	2017	USA	“Barriers to adherence”		●	15
17	AACTG adherence instrument -Supplemented for the Vancouver Injection Drug Users Study (VIDUS)	Kerr et al. 2004 [34]	2004	Canada	“Reasons for missing doses of HAART”^c^		●	13
18	Vancouver Injection Drug Users Study (VIDUS) questionnaire	Kerr et al. 2005 [35]	2005	Canada	“Reasons for discontinuing HAART”		●	15
19	AACTG adherence instrument -Adapted for the Multicenter AIDS Cohort Study (MACS), Medication Adherence Form	Kleeberger et al. 2001 [36]	2001	USA	“Reasons for missing […] medications”^c^	●		15
20	The study to understand the natural history of HIV/AIDS in the era of effective therapy (SUN) study, questionnaire	Kyser et al. 2011 [37]	2011	USA	“Main reason […] for missing medication”		●	6
21	–	Macdonell et al. 2013 [38]	2013	USA	“Barriers to medication adherence”		●	18
22	Community Programs for Clinical Research on AIDS (CPCRA), Antiretroviral Medication Self-Report -Form 646, Version 4, 2003, Section C, Question 2	Mannheimer et al. 2002 [39]	2003	USA	“Reasons why people miss taking their antiretroviral drugs”		●	10
23	AACTG adherence instrument -Adapted	Murphy et al. 2000 [40]	2000	USA	“Barriers to adherence”^c^		●	23
24	AACTG adherence instrument -Supplemented by adolescent-specific issues for the Reaching for Excellence in Adolescent Care and Health (REACH) Project	Murphy et al. 2003 [41]	2003	USA	“Barriers to adherence”^c^		●	19
25	The HIV Epidemiology Research Study (HERS) and Women’s Inter-Agency HIV Study (WIHS), substudy interview instruments	Schuman et al. 2001 [42]	2001	USA	“Reasons that occasionally or frequently interfered with adherence”		●	11
26	US Military HIV Natural History Cohort Study (NHS), HIV Medication Adherence History, Form 168.40.1	n.a.	2010	USA	“Reasons for missed doses”		●	23
27	HIV Medication Self-Reported Nonadherence Reasons (SNAR) Index	Schönnesson et al. 2004 [43]	2004	Sweden	“Reasons for nonadherence to HIV-medication” -Medication concerns and routine disruptions^c^		●	11
28	AACTG adherence instrument -Modified for the New York City Study	Stirratt et al. 2006 [44]	2006	USA	“Reasons for missed ART doses”^c^		●	22
29	–	Walsh et al. 2001 [45]	2001	UK	“Reasons for missing doses”	●		20
30	–	Zorilla et al. 2003 [46]	2003	USA (Puerto Rico)	“Reasons for not taking medications”		●	8
31	–	Zorilla et al. 2003 [46]	2003	USA (Puerto Rico)	“Reasons for taking medications at a different time”		●	7

# = Number assigned to the instrument, as in Table 2

^a^Number of specific items (e.g., does not include space provided for “other” elements not included in the measure)

^b^As indicated by the publications considered in this review (may not be exhaustive)

^c^AACTG adherence instrument or derivative thereof, as reported by developers

### Thematic coverage: instrument breadth and depth

Percent agreement for the item mapping was 88.4%. Thirty-five items were not mapped to the framework. Twenty-three of these, from 5 instruments, concerned “Likely clinically justified reasons” for not taking a specific antiretroviral agent or treatment (e.g., “Recommended by doctor”, “Changing regimens”). These items did not qualify as barriers, as they concerned situations in which the medication no longer seemed clinically indicated. Similarly, 4 other items related to “*How* a person was non-adherent” (e.g., “Doubled up on a dose because you missed a dose”), falling beyond the framework’s scope. Finally, 8 items (/408, 2%) could not be confidently mapped, for lack of clarity (e.g., “You had a bad event happen that you felt was related to taking the pills”).

Table 2 reports the findings on instrument breadth and depth. On average, breadth was 4.4/6 themes (*SD* = 1.2). The majority of instruments covered the broad themes of “Lifestyle factors” (94%), the “Characteristics of antiretroviral therapy” (90%), “Cognitive and emotional aspects” (84%), the “Social and material context” (84%) and the “Health experience and state” (61%). Less than a quarter (23%) covered the “Healthcare services and system” theme. As to depth, it was, on average, 7.0/20 subthemes (*SD* = 3.0). Individual subthemes were addressed in between 3% and 88% of instruments. A majority of instruments contained at least one item on the subthemes of “Demands and organization of daily life” (88%) (e.g., change/break in daily routine, away from home, forgot, fell asleep/overslept, ran out of pills); “Side effects” (81%); “Affect” (71%), especially, feeling depressed/overwhelmed; “Beliefs” about adherence, ART or HIV (63%) (e.g., felt like drug was toxic/harmful); “Instructions” for ART (61%) (e.g., too many pills, problems taking pills at specific times); “HIV stigma and privacy” (61%) (e.g., did not want others to notice); and “Bodily signals” (52%), particularly, feeling sick or ill.

**Table 2:** Thematic coverage of perceived barriers to antiretroviral therapy adherence in the included patient-report instruments

Framework (sub)theme		Instrument^a^																															
		1	2	3	4	5	6	7	8	9	10	11	12	13	14	15	16	17	18	19	20	21	22	23	24	25	26	27	28	29	30	31	%^b^
Cognitive & emotional aspects	-Affect	●	●		●	●	●	●	●		●			●			●	●	●	●		●		●	●	●		●	●	●	●	●	71
	-Beliefs	●	●	●	●	●		●	●		●			●				●	●	●		●	●	●	●	●	●		●	●			63
	-Acceptance					●																●		●									10
	-Motivation																			●	●	●		●			●		●				19
	-Knowledge		●		●				●					●			●					●	●	●	●					●			32
Lifestyle factors	-Demands & organization of daily life	●	●	●	●			●	●	●	●	●	●	●		●	●	●	●	●	●	●	●	●	●	●	●	●	●	●	●	●	88
	-Substance use			●	●	●		●									●	●	●							●	●		●	●			35
Social & material context	-Relations with others		●		●				●	●					●							●				●			●	●			29
	-HIV stigma & privacy	●	●	●	●	●		●	●			●		●			●	●		●		●	●	●	●		●	●	●				61
	-Challenging material circumstances		●			●					●		●		●		●		●	●													26
Characteristics of ART	-Side effects	●	●	●	●		●		●		●	●	●	●	●		●	●	●	●	●	●	●	●	●	●	●		●	●	●		81
	-Instructions	●	●	●	●			●	●		●	●	●	●	●			●		●			●	●	●		●	●	●				61
	-Physical features			●	●																	●			●			●		●			19
Health experience & state	-Bodily signals	●		●	●	●	●	●										●		●		●		●	●	●	●	●	●	●			52
	-Medical signs of HIV/general health			●																											●	●	10
	-Comorbidity							●											●			●											10
Healthcare services & system	-Patient-provider relationship				●																			●									6
	-HIV clinic issues		●																														3
	-Pharmacy issues																●					●											6
	-Health insurance														●							●				●							10
Breadth (/6)		5	5	5	6	4	3	5	5	2	4	3	3	4	3	1	5	5	5	5	3	6	4	6	5	6	5	5	5	5	4	3	
Depth (/20)		7	10	9	12	7	3	8	8	2	6	4	4	7	6	1	8	8	7	9	3	14	6	11	9	8	8	6	10	9	4	3	

Each dot represents the presence of at least one item covering the given subtheme within a specific instrument

^a^Number assigned to the instrument, as in Table 1

^b^Percentage of instruments containing representation of a given subtheme

## Discussion

This review builds on our previous work. It mapped the items of existing HIV-specific measures used in developed countries of patient-reported barriers to ART adherence to our patient-informed conceptual framework. On average, the 31 instruments identified had a conceptual breadth of 73% and a depth of only 35%. Additionally, patient involvement was reported for the development of less than a third of instruments (29%). Together, these findings raise concerns about the content validity of many measures, if they are intended to capture patient perceived ART adherence barriers.

A PROM’s content validity depends, in part, on patient perception of the measure’s comprehensiveness (i.e. the inclusion of all key concepts) [12]. Our findings suggest a disparity between relevant and meaningful adherence barriers for patients (as identified in our previous literature search for our framework [13]), and what the identified instruments are measuring. On the level of broad themes, the least covered, “Healthcare services and system”, was addressed by approximately 1 in 4 instruments. While this was also the least common theme in the qualitative studies contributing to the framework, two-thirds of them referred to it, especially to the “Patient-provider relationship” subtheme, described later. Among its other subthemes are “Health insurance” and “Pharmacy issues” (e.g., trouble going to the pharmacy; getting timely refills, for instance, due to stockouts). In resource rich settings, many PLHIV can have difficulty covering their pharmacy dispensing costs and travel costs to the clinic, with research suggesting that this financial stress is infrequently addressed in HIV care and associated with interrupting and ceasing ART [47]. Furthermore, “Health care team and system-related factors” is a major dimension of the World Health Organization model of factors that affect adherence in chronic conditions, including HIV [48]. Hence, it appears this theme requires representation in a comprehensive measure of ART adherence barriers.

On the level of subthemes, other significant disparities were apparent. While 54% of studies informing the framework mentioned the barrier of “Acceptance”, that is, non-acceptance, denial or avoidance of one’s HIV diagnosis, this was addressed in only 10% of measures. Furthermore, over three-quarters (76%) of studies mentioned “Relations with others” as a barrier, most frequently, inadequate social support and relationship-related problems or stress. By comparison, only 29% of instruments contained any item on this subtheme. As a final example, while 59% of studies portrayed the “Patient-provider relationship” as a barrier, particularly in terms of mistrust of the provider, provider negativity/lack of supportiveness, poor communication, and feelings of coercion/powerlessness, this subtheme was apparent in only 2 instruments/2 items.

Overall, our findings suggest that no measure of perceived barriers to ART adherence, as defined, may sufficiently capture this concept. A lack of comprehensiveness has implications for our understanding of the ART adherence barriers experienced by PLHIV, the estimation of their prevalence, and, ultimately, the design of patient- centered interventions to address them. The infrequent patient involvement observed in the measures’ development may offer some explanation, if reported involvement reflects actual involvement.

This review is limited by the search strategy employed; we did not attempt to locate all existing instruments, instrument versions or validation studies per instrument. No data was extracted on the measures’ psychometric properties which may shed further light on the findings. Nevertheless, the results presented support the development of our PROM, the content of which will be evaluated by PLHIV and providers in Canada and France with online Delphi techniques [49].

## Abbreviations

AACTG: Adult AIDS Clinical Trial Group
AIDS: Acquired Immune Deficiency Syndrome
ART: Antiretroviral therapy
HIV: Human immunodeficiency virus
PLHIV: People living with HIV
PROM: Patient-reported outcome measure
